# Combining bird tracking data with high-resolution thermal mapping to identify microclimate refugia

**DOI:** 10.1038/s41598-023-31746-x

**Published:** 2023-03-23

**Authors:** Rita F. Ramos, Aldina M. A. Franco, James J. Gilroy, João P. Silva

**Affiliations:** 1 CIBIO/InBIO, Centro de Investigação em Biodiversidade e Recursos Genéticos Laboratório Associado, Universidade do Porto, Campus Agrário de Vairão, 4485-661 Vairão, Portugal; 2 CIBIO/InBIO, Centro de Investigação em Biodiversidade e Recursos Genéticos Laboratório Associado Instituto Superior de Agronomia, Universidade de Lisboa, Tapada da Ajuda, 1349-017 Lisboa, Portugal; 3 Departamento Biologia Faculdade de Ciências, Universidade do Porto, Vairão, Portugal; 4 BIOPOLIS Program in Genomics, Biodiversity and Land Planning, CIBIO, Campus de Vairão, 4485-661 Vairão, Portugal; 5 School of Environmental Sciences, University of East Anglia, Norwich, UK

**Keywords:** Biodiversity, Climate-change ecology, Conservation biology, Ecological modelling, Ecosystem ecology

## Abstract

Elevated temperatures can have a range of fitness impacts, including high metabolic cost of thermoregulation, hence access to microclimate refugia may buffer individuals against exposure to high temperatures. However, studies examining the use of microclimate refugia, remain scarce. We combined high resolution microclimate modelling with GPS tracking data as a novel approach to identify the use and availability of cooler microclimate refugia (sites > 0.5 °C cooler than the surrounding landscape) at the scales experienced by individual animals. 77 little bustards (*Tetrax tetrax*) were tracked between 2009 and 2019. The 92,685 GPS locations obtained and their surrounding 500 m areas were characterised with hourly temperature and habitat information at 30 m × 30 m and used to determine microclimate refugia availability and use. We found that the semi-natural grassland landscapes used by little bustards have limited availability of cooler microclimate areas—fewer than 30% of the locations. The use of cooler microclimate sites by little bustards increased at higher ambient temperatures, suggesting that individuals actively utilise microclimate refugia in extreme heat conditions. Microclimate refugia availability and use were greater in areas with heterogeneous vegetation cover, and in coastal areas. This study identified the landscape characteristics that provide microclimate opportunities and shelter from extreme heat conditions. Little bustards made greater use of microclimate refugia with increasing temperatures, particularly during the breeding season, when individuals are highly site faithful. This information can help identify areas where populations might be particularly exposed to climate extremes due to a lack of microclimate refugia, and which habitat management measures may buffer populations from expected increased exposure to temperature extremes.

## Introduction

Microrefugia are localised areas of climatic stability where species can persist under climate change^1^. Microrefugia may be experienced by individuals at the meter scale or smaller^2^^.^ In contrast, macrorefugia is measured at scales of hundreds of kilometres^3,4^. Many species use microclimate refugia daily, either to rest, or to avoid periods of high temperatures during the day or low temperatures during the night^6,7^. This dynamic temporal use of microclimate refugia enables species to persist in areas where environmental conditions fluctuate or become unsuitable in parts of the day or the year^8,9^. Thus, microclimate refugia use may allow populations to persist in areas where larger-scale climate conditions are becoming unsuitable.

Use of microrefugia by individual animals is still poorly understood, especially for large mobile taxa. However, the combination of new tracking technologies and novel tools for temperature modelling at finer scales^5^ is opening up new avenues for research, enabling researchers to quantify the importance of small-scale climate refugia for population persistence under climate change.

Recent studies modelling microclimate at a fine scale have demonstrated how habitat structure and topography can be combined to provide significant microclimate refugia, with ground-level temperatures varying by up to 5 °C across scales of a few meters in some landscapes^2,4^. This variation has important consequences for local-scale species occurrence patterns. For example, in Meadow pipits (*Anthus pratensis*) microclimate conditions had a stronger effect than macroclimate and accounted for approximately a third of the variation in occupancy probability across the United Kingdom^4^. These findings highlight the importance of microclimate in facilitating species persistence in areas where macro-scale conditions may not (or no longer) be suitable. Microclimate refugia may be particularly critical for population persistence at the edge of species ranges^10,11^ or in areas predicted to be exposed to high variability due to climate change^12^.

Mediterranean ecosystems, like the Iberian Peninsula in western Europe, are among the world’s most vulnerable ecosystems to climate change^12^ and are expected to continue to suffer from extensive warming and increasing drought and heat wave frequency^13^. Climatic conditions are expected to become particularly extreme in flat, open areas with low vegetation cover, as is the case of semi-natural grasslands. Microclimate refugia may therefore be crucial for the persistence of many endangered species in the Mediterranean region, but our understanding of the characteristics of important microrefugia remains limited particularly for large mobile taxa.

This study examines availability and use of microclimate refugia using the little bustard, *Tetrax tetrax* as a case study. This grassland specialist is classified as ‘Near Threatened’^14^ and the Iberian Peninsula is considered the stronghold of the species’ European distribution, however, recent studies indicate a severe decline of breeding numbers in the region, despite conservation efforts^15,16^.

As a grassland specialist inhabiting open areas with short vegetation, the little bustard is a good model species to understand use of microclimate refugia in landscapes with limited refugia opportunities that are warming due to climate change. Research have showed that little bustard daily activity levels decrease when temperatures exceed 25°C^17^, which are frequent both in breeding and post-breeding seasons. In addition, as shown for other bird species, little bustards are likely to experience thermal stress above approximately 37 °C, and lethal body temperature at 46°C^18–20^. We hypothesis that little bustards use microclimate refugia to avoid detrimental effects of exposure to thermic stress. We test the prediction that increasing exposure to high temperatures, particularly during the breeding and post-breeding seasons, will require little bustards to make more use of microclimate refugia (where available) to minimise the impacts of thermal stress.

We used long-term high-resolution GPS-tracking data to quantify microclimate refugia use across a large area of the little bustard’s western European range. Our objectives are to: (i) identify microclimate refugia using high spatial and temporal resolution environmental and tracking data, (ii) determine if the use of microclimate refugia increases with exposure to high temperatures, and (iii) identify the characteristics of landscape areas that provide microclimate refugia opportunities.

## Results

### Identifying microclimate refugia availability and use

**Figure 1:**
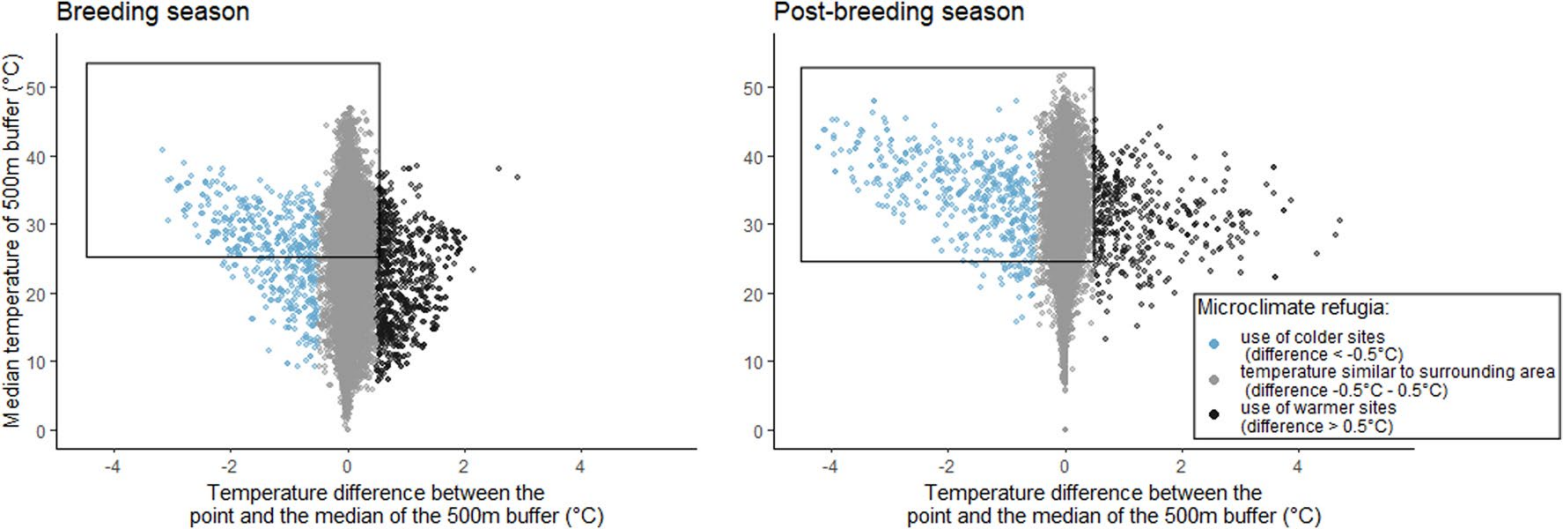
Difference between the temperature of the little bustard locations and the median temperature of the buffer area, for both breeding and post-breeding seasons. The black squares identify the GPS location data used to understand microclimate refugia availability and use by little bustards. Only locations obtained at ambient temperature above 25 °C are included in the analysis where sites with and without cooler microclimate refugia opportunities are compared.

The dataset included 43,500 GPS locations obtained during the breeding season and 49,185 locations in post-breeding (Fig. 1, SI1). In total we obtained 102 bird/year of data in the breeding season and 90 bird/years in the post-breeding. Most of the GPS locations (97% for breeding and 98% for post-breeding seasons) were within − 0.5 and 0.5 °C of the median temperature of the 500 m surrounding landscape (Fig. 1), indicating little bustards use areas with similar temperatures to their surroundings.

**Figure 2:**
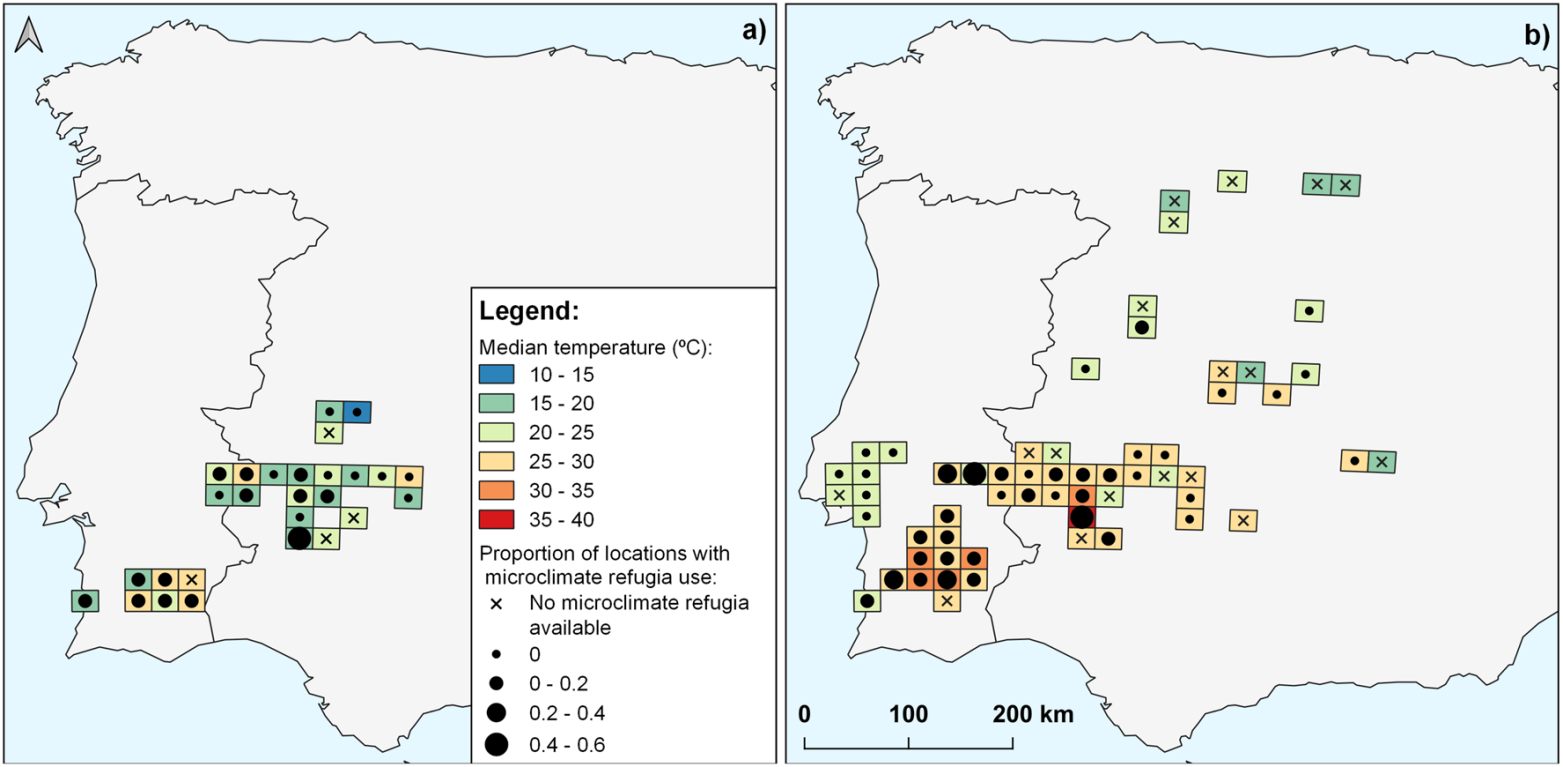
Mean median temperature for little bustard locations in the 25 × 25 km grid cells occupied and proportion of locations where microclimate refugia was used by little bustards (circles), in both a) breeding and b) post-breeding seasons. The “x” shows the grids were there was no microclimate refugia available. Map made with QGIS 3.28.2 (www.qgis.org).

Half of the little bustard locations (35% in the breeding and 63% in the post-breeding) were at ambient temperatures above 25 °C, and approximately a quarter of the GPS locations (26.7% of breeding and 29.5% of post-breeding) were identified as having microclimate refugia available within the buffer area (Table 1, Fig. 2). At very high temperatures (above 37 °C), microclimate refugia were available within 500 m of 24.9% and 36.2% of the little bustard GPS locations obtained for breeding and post-breeding seasons, respectively (Table 1). Microrefugia sites provided temperatures up to 4 °C cooler than the surrounding landscape and the largest temperature differences obtained were for locations at more than 40 °C and during the post-breeding season.

**Table 1:** Summary information of microclimate refugia availability in the buffer area and use by little bustard at above optimal temperature (25–37ºC) and at thermal stress temperature (> 37 °C), for both breeding and post-breeding seasons (Fig. 2).

Temperature (ºC)	Breeding			Post-breeding		
	Total (%)	Availability (%)	Use (%)	Total (%)	Availability (%)	Use (%)
25–37 ºC	14,491 (94.9%)	3,876 (26.8%)	291 (7.5%)	25,861 (82.9%)	7,286 (28.2%)	264 (3.6%)
≥ 37 ºC	780 (5.1%)	194 (24.9%)	5 (2.6%)	5,337 (17.1%)	1,933 (36.2%)	158 (8.2%)
Total	15,271 (100%)	4,070 (26.7%)	296 (7.3%)	31,198 (100%)	9,219 (29.5%)	422 (4.6%)

The temporal use of microclimate refugia was low (7.3% of breeding and 4.6% of post-breeding locations where microclimate refugia were available), but peaked during the post-breeding season when ambient temperatures exceeded 37 °C (use at 8.2% of the locations) (Table 1, Fig. 2).

### Determinants of microclimate refugia availability

**Figure 3:**
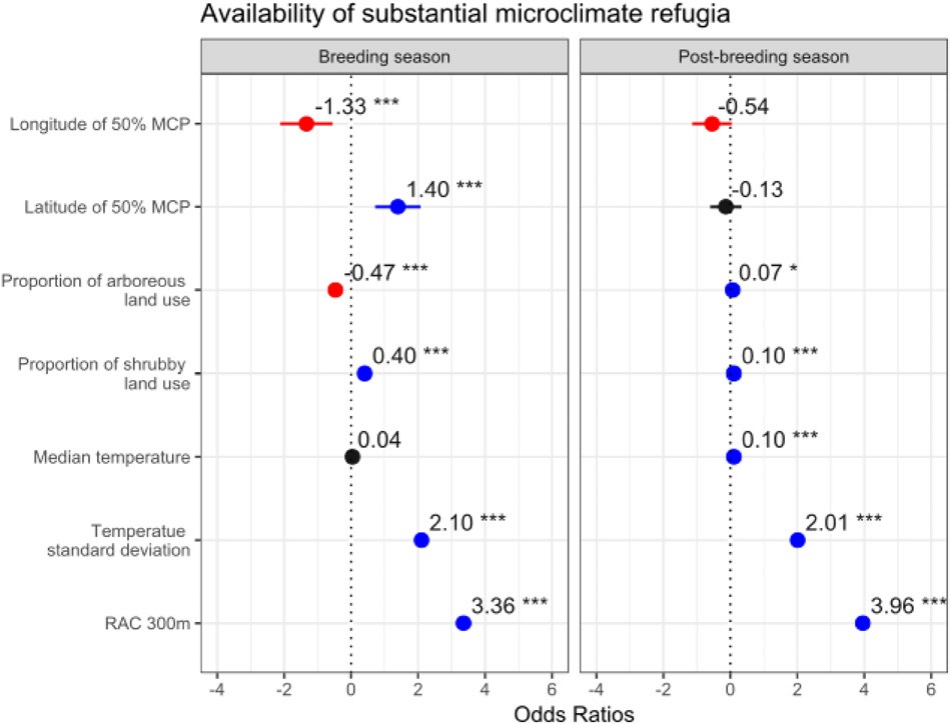
GLMM results for the predictors of availability of microclimate refugia, for both breeding and post-breeding seasons. Variable significancy is shown: *** *p* < 0.01; * 0.01 < p < 0.05; others, p > 0.05. Positive effects are shown in blue, negative effects in red and not significant effects in black.

During the breeding season, availability of microclimate refugia was negatively associated with longitude (F = − 1.33, p < 0.01) and positively associated with latitude (F = 1.40, p < 0.01) indicating general trends towards higher availability of microclimate refugia in areas occupied by little bustards within northern and western (Fig. 3, SI2). Microclimate refugia availability was negatively associated with proportion of arboreous land use within the buffer area (F = − 0.47, p < 0.01) and positively associated with proportion of shrubby land use (F = 0.40, p < 0.01) (Fig. 3, SI2). In the post-breeding, microclimate refugia availability was positively associated with higher proportions of both shrubby and arboreous land uses (F = 0.10, p < 0.01 and F = 0.07) (Fig. 3, SI2).

At higher ambient temperatures in the post-breeding season, little bustards used sites with greater microclimate refugia availability (F = 0.11, p < 0.01). Locations with higher availability of microclimate refugia also showed greater temperature standard deviations across the 500 m area surrounding the bird locations, for both breeding (F = 2.10, p < 0.01) and post-breeding seasons (F = 2.01, p < 0.01) (Fig. 3, SI2).

### Determinants of microclimate refugia use

**Figure 4:**
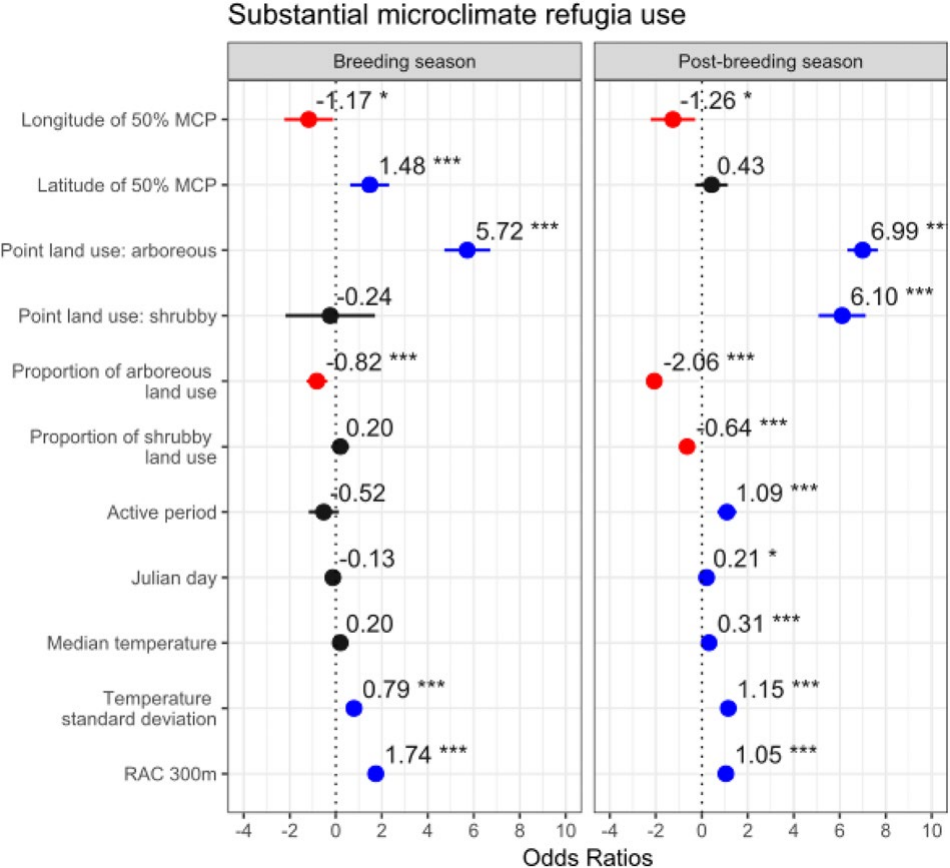
GLMM results for the predictors of microclimate refugia use, for both breeding and post-breeding seasons. Variable significancy is shown: *** p < 0.01; * 0.01 < p < 0.05; others, p > 0.05. Positive effects are shown in blue, negative effects in red and not significant effects in black.

The use of microclimate refugia was negatively associated with longitude, for both breeding and post-breeding seasons (F = − 1.17, p = 0.03; F = − 1.26, p = 0.01, respectively), and was positively associated with latitude during breeding season (F = 1.48, p < 0.01), indicating higher microclimate refugia use in more northern and coastal areas of the Iberian Peninsula (Fig. 4, SI3), mirroring patterns for microclimate refugia availability.

Microclimate refugia use was positively associated with arboreous locations in both seasons (F = 5.72, p < 0.01, for breeding; F = 6.99, p < 0.01, for post-breeding), as well as with shrubby locations during post-breeding (F = 6.10, p < 0.01) (Fig. 4, SI3). Additionally, it was negatively associated with high proportions of arboreous land use in the surrounding 500 m in both seasons (F = − 0.82, p < 0.01, for breeding; F = − 2.07, p < 0.01, for post-breeding), as well as with high proportions of surrounding shrubby land use, during post-breeding (F = − 0.64, p < 0.01). Sites that were used as microclimate refugia therefore tended to be focal locations with some arboreous or shrubby cover sitting within areas dominated by other more open habitats.

In the post-breeding season, microclimate refugia use was positively associated with the little bustard active period (F = 1.09, p < 0.01) and the Julian day (F = 0.21, p = 0.02) (Fig. 4, SI3).

Lastly, little bustard use of microclimate refugia during post-breeding was positively associated with the median temperature of the surrounding area (F = 0.31, p < 0.01), and with the temperature standard deviation, for both breeding (F = 0.79, p < 0.01) and post-breeding seasons (F = 1.15, p < 0.01) (Fig. 4, SI3).

## Discussion

**Figure 5:**
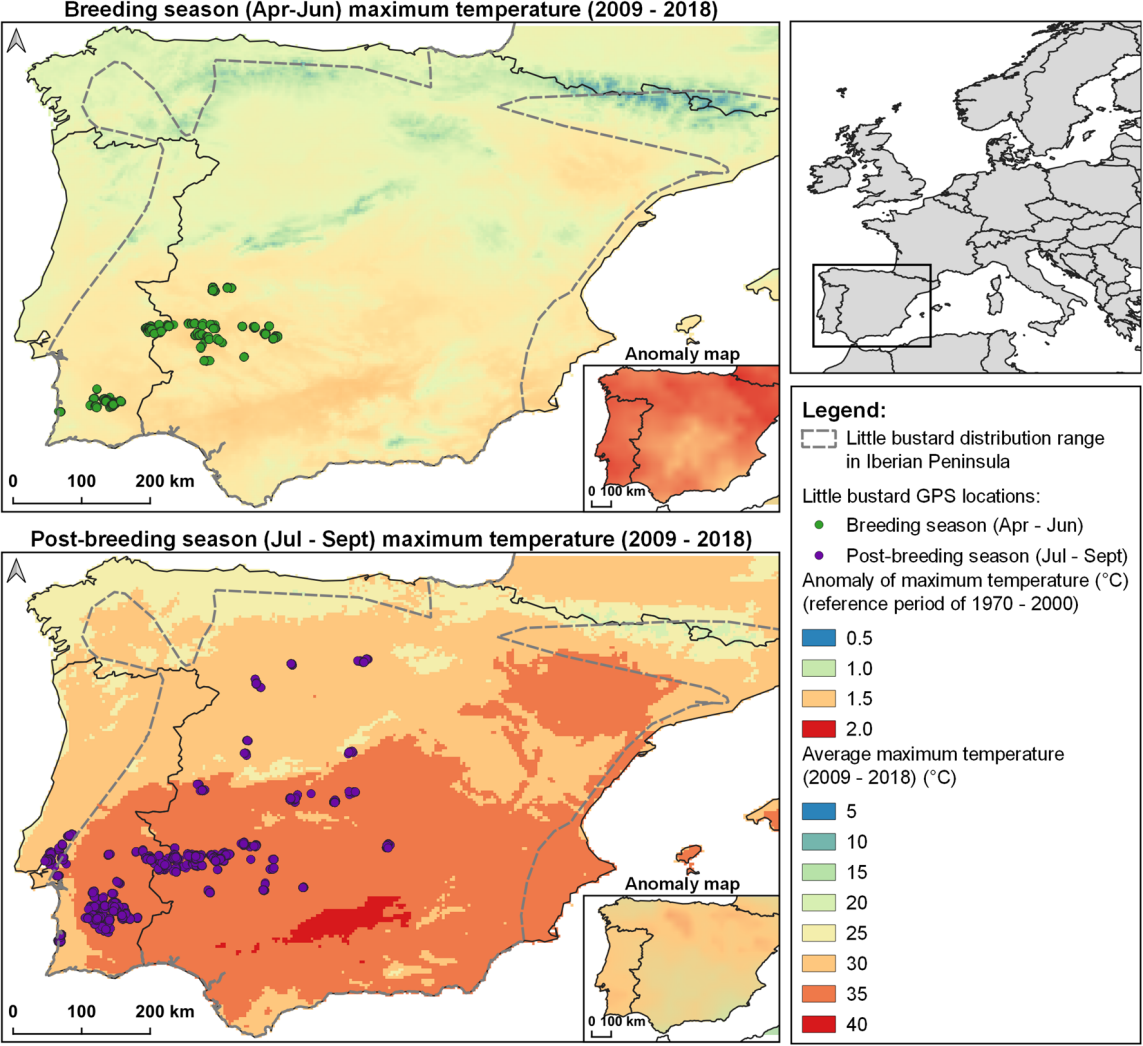
Clusters of little bustards’ GPS locations displayed over the average daily maximum temperature between 2009 and 2018 for breeding season (April–June) and post-breeding (July–September). Corresponding anomaly maps using the reference period of 1970–2000 are shown. Dashed grey line represents the little bustard distribution in the Iberian Peninsula^47^. Temperature data obtained from the WorldClim^46^. Map made with QGIS 3.28.2 (www.qgis.org).

Our 11-year tracking dataset reveals the use and availability of microclimate refugia of a threatened grassland species in areas exposed to high and increasing temperatures, extreme drought, and frequent heat waves^12^. We find that little bustards use microclimate refugia in both breeding and post-breeding seasons, especially when exposed to higher temperatures. The microclimate refugia sites used are characterized by small patches of shrubs and trees in an herbaceous landscape, which promotes heterogeneous temperatures. This heterogeneous thermal landscape can mitigate against detrimental effects of increasing temperatures observed across the Iberian Peninsula over the past 30 years (Fig. 5) which are expected to continue under climate change^12^. Additionally, microclimate refugia can shelter individuals from high temperatures during critical parts of the annual cycle^21^. Nonetheless, further research on the physiological, behavioural and fitness consequences of exposure to temperature extremes is needed to ascertain the extent to which microclimate refugia availability may provide effective conservation measures.

Birds can use thermoregulation mechanisms (e.g. evaporating water through the skin) and behavioural changes (e.g. decreasing activity levels when temperatures are higher) to respond to temperature extremes, but all these can impact their fitness^17,21,22^. The apparent use of microclimate refugia by little bustards was low in comparison to its availability. Nonetheless the use increased when ambient temperature rose. The percentage of use of microclimate refugia above 37 °C was greatest during post-breeding (8.2%) when ambient temperatures tend to peak, and when the birds are not actively breeding, and therefore are less site faithful. Little bustards show a strong site fidelity during the breeding season, even when their display areas undergo major land use changes^23,24^. Males remain in their display areas characterized by low vegetation cover^25^, making this species particularly vulnerable to temperature extremes. Although in our study, temperatures above 37 °C during breeding season represent only 5% of the data, the higher temperature anomalies registered during this period (Fig. 5), suggest that microrefugia may play an important role in buffering high temperatures. This buffering effect, reduces the risk of overheating and costly metabolic functions to thermoregulate, and consequently may prevent fitness depletion^26^.

Little bustards were often exposed to temperatures above 25 °C in the post-breeding, the temperature above which little bustards drastically reduce their activity patterns^17^. In the summer months (post-breeding season) temperatures often exceeded 40 °C. This was associated with an increase in the use of microclimate refugia which was observed at very high temperatures (> 37 °C).

An alternative strategy many individuals adopt to cope with extreme temperatures is to migrate to milder areas in the Iberian Peninsula. The little bustard is a partial-migratory species, with most Iberian individuals performing short- to medium-distance movements during post-breeding^27^. These milder areas are mainly located at the northern range of the species’ Iberian distribution and in coastal areas (e.g., estuaries).

This movement strategy enables individuals to avoid the extreme temperature conditions experienced in some post-breeding areas. Northern and Western areas can be up to 10 °C colder than inland southwestern areas, and thus can act as macro-scale refugia. This may help explain why, despite an increase in microclimate refugia use above 37 °C, the overall (above 25 °C) percentage of microclimate refugia use in the post-breeding season decreases. During post-breeding, little bustards are less dependent on grasslands, and can use areas with different land uses where they may be less exposed to high temperatures, such as irrigated fields and olive groves with more heterogeneous temperatures^28^. Perhaps unsurprisingly, we found that microclimate refugia availability was higher in areas with more shrubby or arboreous cover during the post-breeding, which correspond to the non-grassland areas little bustards' resort to during the post-breeding season. By contrast, during the breeding season, little bustards are restricted to sites that maximise their ability to be seen by conspecifics^17^. This need to be conspicuous and strong site fidelity behaviour to lekking areas^29^ likely exposes little bustards to elevated temperatures in the breeding season.

This season is also the period with the highest temperature anomalies, hence if this trend continues, availability of small-scale microclimate refugia may become even more important in the future due to climate change.

Our results suggest that the presence of small patches of trees and shrubs within landscapes dominated by herbaceous vegetation can provide microclimate refugia, as even in smaller patches the canopies’ shade allows for lower temperatures^30^. Furthermore, the heterogeneous thermal landscape that is created by shaded areas protects our study species from exposure to high temperatures as well as insects that the little bustard may consume^31,32^. This is an indirect positive effect of microclimate refugia availability, that benefits this endangered species.

Both microclimate refugia availability and use increase in northern and western areas of southwestern Iberia during breeding season. The southern populations in the Baixo Alentejo (Portugal) region have little microclimate refugia overall, despite being one of the species’ most important breeding areas in Iberia^25,33^. Landscapes there are less topographically diverse and more dominated by open areas than other parts of the range^34^, offering fewer microclimate refugia.

Microclimate refugia availability was higher in both seasons when temperatures in the 500 m buffer area were more heterogenous. These temperature-heterogeneous areas may allow little bustards to have quick access to cooler areas that enable them to reduce metabolic heat production and to thermoregulate^19^.

In the post-breeding, little bustards used more microclimate refugia during their active period of the day and towards the end of the post-breeding season. These results may be explained by high temperature variation during the active period, as the temperature rises at different rates from early morning until the middle of the day and decrease at different rates in the second part of the day. Additionally, in the Iberian Peninsula, temperatures can reach 40 °C during the post-breading season (between July and August). These periods of high temperature in the middle of the day correspond to little bustards’ inactive period^17^.

Lower refugia use during the inactive period suggests that little bustards may be adopting other strategies to cool down. For instance, they may be keeping activities to a minimum to reduce metabolic heat production or using behavioural thermoregulation^6,22^. Additionally, areas with microclimate refugia may have increased foraging resource availability^31,32^ and thus are more used in the active period.

### Methods limitations

The *microclima* package is the best available resolution temperature modelling tool, but it still has important limitations. It uses a relatively simplistic schema for vegetation structure shading effect and does not account for the influence of drought on soil heating^20^, this will likely lead to underestimates of temperature in our Mediterranean study area. Moreover, the spatial scaling of our modelling approach may have underestimated the use of microclimate refugia in the case of very small-scale habitat features. Individuals experience their thermal environment at scales from centimetres to few meters^3^, and thus might utilise small, isolated trees or bushes as microclimate refugia not captured by the land cover resolution (100 m)^35^. The technology needed to obtain such fine-scale vegetation information across a species’ distribution is not yet fully available, though increases in LiDAR coverage and decrease of acquisition costs may enable this in the future for relatively large landscape scales^36,37^.

We focussed on quantifying microclimate refugia in areas where little bustards were present, and thus did not investigate potential refugia availability in the wider landscape. However, we quantified microclimate refugia availability within a 500 m buffer, which represents a large area compared to the range used by territorial males and their average distance moved during the breeding season^29^ (see SI4). Moreover, although microclimate refugia use is limited in both seasons, the results show that a quarter of little bustard locations in the breeding season and more than a third in the post-breeding season are in areas where microclimate refugia is available within 500 m.

Microclimate refugia availability is a fine scale metric that considers the availability of colder sites in the 500 m buffer around each GPS location.

Finally, this study focuses on understanding the factors that determine the availability and use of microclimate refugia and does not evaluate whether these microclimate refugia prevent exposure to thermal stress.

Future studies examining microclimate refugia opportunities at the wider landscape scale could be informative in identifying areas where refuge availability may be limiting, particularly if parts of the former range have already become unsuitable for little bustard presence due to the lack of microclimate buffering opportunities.

Finally, we were only able to capture and track male bustards in our study system, and our findings are thus specific to males. Given the reproductive strategy of the species, female decisions on microhabitat selection are likely to differ markedly from those of males during the breeding period, reflecting the constraints imposed by nesting and chick-rearing. During post-breeding, however, little bustards form mixed-sex flocks and thus our male-biased sample may be more representative of the behaviour of both sexes during that period.

## Conclusions

We show that tracking data and microclimate modelling tools can yield important insights into the availability and use of microclimate refugia. These results can be used to identify the habitat features that provide microclimate refugia opportunities for populations at the edge of species’ ranges or those affected by climate warming and exposure to extreme temperatures. Our results show that the presence of small patches of taller non-herbaceous vegetation, provide microclimate refugia opportunities through the creation of a more heterogeneous thermal landscape^30,38^. Microclimate refugia were available in approximately a quarter of the little bustard locations, suggesting that availability of microclimate refugia may be limited for these grassland birds in some parts of their Iberian range (e.g., Baixo Alentejo, Portugal).

Further research on the contribution of microclimate refugia for species fitness, behaviour and habitat selection is still needed to inform the design and management of breeding and post-breeding areas resilient to climate warming.

## Materials and methods

### Study area and study system

Between 2009 and 2019, 77 male little bustards were captured and tagged in five breeding areas across the Southwestern Iberian Peninsula, in Alentejo (Portugal) and Extremadura (Spain) during the breeding season (April and May; Fig. 5). In this area of Europe, temperatures often exceed 37 °C and heat waves have increased in frequency in the last three decades^39^. Little bustards breed in an exploded lekking system^40^, which consists in males defending their territories from other males in wide areas while showing an exuberant displaying behaviour to attract visiting females and mate (for more details see Morales et al. 2001). Once breeding is completed, most little bustards migrate to post-breeding areas scattered across Iberia.

Breeding males were captured using decoys and snares at the start of the breeding season. Although some females were attracted to the decoy, they were not the targeted sex, due to different behaviours, which require more complex capture techniques. Males were attracted by a stuffed female acting as a decoy and trapped with snares, placed around the decoy^41^. GPS tracking devices varied between 2 and 4% ($\overline{x }$ = 3.2%) of the birds’ mass^42^, were deployed using a thoracic harness made of Ribbon Teflon with a weak link to avoid lifelong deployment.

Two types of Solar GPS devices were used. Platform Transmitter Terminal (Solar Argos/GPS 30 g PTT—Microwave Telemetry) were deployed on 28 birds between 2009 and 2011, and Global System for Mobile Communications (GSM) devices (Flyway 38 g—Movetech Telemetry) were deployed in 49 birds between 2014 and 2019. Transmitters were programmed to record a GPS position every 2 h (PPT) or 10 to 30 min (GSM). Bird trapping and GPS tagging were approved by the Instituto da Conservação da Natureza e das Florestas (Portuguese Government agency responsible for Wildlife and Forests Management and Conservation) through licenses to João Paulo Silva (ICNF/CAPT/2014, ICNF/CAPT/2015) and Consejería de Medio Ambiente y Rural, Políticas Agrarias y Territorio of the Junta de Extremadura (Spanish Ministry of Environment and Rural, Agrarian Policies and Territory of the Extremadura region) through the license to José Mª Abad-Gómez.

We filtered the GPS dataset to only include locations on the ground with null velocity, removing all in-flight fixes. The dataset was then subdivided into two seasons—breeding and post-breeding – the temporal limits of which were defined for each breeding area independently, as the breeding phenology and movement dates of the little bustards to post-breeding areas vary geographically. We defined the start of the breeding season uniformly as the 1^st^ of April and defined the end of the breeding season for each breeding areas as the day the first tracked male moved from that area towards its post-breeding area, ensuring we captured the core period of the breeding season while avoiding capturing migratory movements and stopover sites^43^. The post-breeding season was defined uniformly for all breeding areas, from 15^th^ of July to 15^th^ of September, representing the hottest period of the year (Fig. 5) when exposure to extreme heat is highest, and food shortages occur^44^. The dates selected for two seasons guaranteed temporal independence as there was no overlap between breeding and post-breeding seasons across all birds.

The highest temperatures recorded in Iberia occur in the southwestern region which coincides with the little bustard Iberian population stronghold^15,48^. The landscape is characterised by semi-natural grasslands created and maintained by agricultural activities and livestock grazing and are thus dependent on human management of the landscape^49^.

During the 11-year period the birds were tracked, the average maximum air temperatures varied between 6.8–28.7 °C in the breeding season (April to June) and 15.8–36.3 °C in the post-breeding (July to September). Relative to a reference period of 1970 – 2000, there was a 1 to 2 °C increase in temperature across the whole region (Fig. 5), with greatest increases during the breeding season (1.5 to 2 °C; Fig. 5)^45,46^.

### Quantifying micro-climatic conditions

We estimated the hourly temperature at 20 cm above ground level for all bird GPS locations using the R packages *microclima*^5^ and *NicheMapR*^50,51^. Microclimate temperature account for the effects of net radiation, coastal influence and cold air drainage into consideration (from *NicheMapR*). These variables are determined at microscale accounting for terrain information such as slope, aspect and hill shade, and canopy shading effects resulting from different habitat types as defined by the International Geosphere-Biosphere Program (IGBP) (from *microclima*). We used the fully automated model, where National Centers for Environmental Prediction^52,53^ (NCEP) climate reanalysis data is downscaled and interpolated to provide hourly information on the reference temperatures and atmospheric forcing conditions that are used to parameterise the model.

We used 30 m × 30 m resolution habitat information from CORINE Land Cover map 2018^35^ to account for canopy shading effects, matching CORINE habitat categories to their equivalent MODIS / IGBP categories using the schema provided in SI5^54^.

For each GPS location we retrieved the temperature information at the focal point, as well as for the surrounding area of 500 m (mapped at 30 × 30 m resolution), for the hour at which the GPS location was obtained. We used the 500 m area (here onwards called buffer area) to evaluate how climatically different a bird’s location was from its nearby surroundings at each point in time using this distance threshold as across the annual cycle, little bustards typically move less than 300 m between two consecutive hourly points (SI4).

### Classifying habitat conditions

We mapped habitat conditions across each buffer area using the 2012 and 2018 CORINE land cover maps^35,55^, selecting the 2012 map for GPS locations between 2009 and 2014, and the 2018 map for locations from 2015 onwards, to account for land use changes that may have occurred during the eleven-year tracking period (see SI6).

To capture habitat variation relevant to little bustards, we simplified the CORINE land cover classes into four categories: herbaceous, shrubby, arboreous vegetation and “other land uses” (this includes urban areas, roads and water bodies). The study area is dominated by herbaceous vegetation^49^. A complete description of CORINE land cover correspondence classes (see SI6). In addition to extracting the land use information at the GPS location point, we also calculated the percentage of each type of land use in the buffer area.

### Defining microclimate refugia

We defined microclimate refugia as locations that were at least 0.5 °C cooler than the median temperature of the surrounding 500 m buffer area. This half degree difference reflects the magnitude of variation that is likely to be perceived by animals^38^, birds in particular, and lead to significant changes in behaviour, thermoregulation and survival^22,38^. We determined the availability of microclimate refugia around each GPS fix (obtained above 25 °C), by calculating the difference between the minimum and the median temperatures within the 500 m buffer. Microclimate refugia was considered to be available when this difference exceeded 0.5 °C. We considered that little bustards were using microclimate refugia when the focal location was at least 0.5 °C cooler than the median temperature of their surrounding buffer area.

### Statistical analysis

We used Generalized Linear Mixed Models^56^ (GLMM) with a binomial error distribution and a logit-link function^57^, to analyse the environmental characteristics of sites that provide microclimate refugia (i.e. their availability was coded as 1 or 0 depending on the presence of refugia within the 500 m buffer), and how environmental conditions influence the probability that refugia are used by little bustards (coded as 1 or 0 depending on whether the focal point was > 0.5 °C cooler than the median of the buffer). We modelled breeding and post-breeding seasons separately, resulting in four GLMMs.

We hypothesised that both availability and use of refugia may vary seasonally, so we included Julian day as an independent variable. Moreover, little bustards’ daily activity patterns may influence refugia use, so we included a binary variable representing active or inactive periods during daytime hours, which have been previously established for the species using tracking data^17^. The inactive periods were between 11 am to 4 pm during the breeding season and between 10 am to 4 pm in the post-breeding season, and we expected birds to be more likely to use microrefugia in the inactive periods.

We also included the longitude and latitude coordinates of each individual’s core area in each season determined by the centroid of the 50% Minimum Convex Polygon (MCP)^42,58^. This was done to account for larger-scale gradients of environmental variation across the Iberian Peninsula (Fig. 5).

For each of our four models (microclimate refugia availability and use, for both breeding and post-breeding seasons), we included variables capturing variation in temperature and habitat, as spatial and temporal thermal variability (Table 2). We tested for multicollinearity between variables, aiming for − 0.7 > r < 0.7 and a variance inflation factor (VIF) smaller than 3^57^. A high negative correlation between the proportion of herbaceous and arboreous habitat was detected, and of these we chose to use the proportion of arboreous habitat in the models.

**Table 2:** Explanatory variables used in the models of microclimate refugia availability and use, for both breeding and post-breeding seasons.

Variable name	Description
Temp. median buffer (ºC)	Median of the temperature data in the buffer area
Temp. SD buffer (ºC)	Log (+ 1) transformation of the standard deviation of the temperature data in the buffer area
Hour	Hourly period (category), either active or inactive^15^
Julian day	Julian day of the GPS locations point
Point land use	Land use of the location (category), either herbaceous, arboreous or shrubby
Prop. herbaceous	Arcsine (square root) transformation of the proportion of herbaceous land use in the buffer area
Prop. arboreous	Arcsine (square root) transformation of the proportion of arboreous land use in the buffer area
Prop. shrubby	Arcsine (square root) transformation of the proportion of shrubby land use in the buffer area
50 MCP long	Longitude of the centroid of the 50% MCP of each individual per year
50 MCP lat	Latitude of the centroid of the 50% MCP of each individual per year

We also used individual ID nested within year as a random factor (with 103 and 90 groups for the breeding and post-breeding seasons, respectively). We filtered the data to only include GPS locations with temperatures above 25 °C prior to modelling^17^, and only included daylight locations between 6 am and 5 pm (breeding) and 7 am and 8 pm (post-breeding) to avoid capturing nocturnal roost sites that could have microclimate characteristics.

To account for the potential spatial autocorrelation (expected in spatially clustered data), we introduced a spatial autocovariate term (RAC)^59,60^ with a neighbourhood of 300 m, calculated from the residuals of a GLMM including all other explanatory variables (i.e., the global model). By using the RAC term, we account for autocorrelation in the response variable, only after fitting the other explanatory variables first^60^. RAC was calculated using the *autocov_dist* function from the *spdep* package in R^61^.

The models including the RAC term were compared against both the null and global model using Akaike’s Information Criterion and ANOVA test^62^ (AIC). The fit of models was evaluated using the Area Under the Curve (AUC)^63^.

All models and summary statistics were run in the R version 3.6.2^64^ in the *lme4* package^65^.

### Approval for animal experiments

Licences to catch and deploy the tracking devices were provided by Conservação da Natureza e das Florestas (Portuguese Government agency responsible for Wildlife and Forests Management and Conservation) through licenses to João Paulo Silva (ICNF/CAPT/2014, ICNF/CAPT/2015) and Consejería de Medio Ambiente y Rural, Políticas Agrarias y Territorio of the Junta de Extremadura (Spanish Ministry of Environment and Rural, Agrarian Policies and Territory of the Extremadura region) through the license to José Mª Abad-Gómez. All captures of little bustards and deployment of the tracking devices were performed in accordance with relevant guidelines and regulations.

## Supplementary Information

Supplementary Information.

## Data Availability

The datasets used and analysed during the current study are available from the corresponding author on reasonable request.
