# Supplementary material for: Combining bird tracking data with high-resolution thermal mapping to identify microclimate refugia
Source: Sci Rep. 2023 Mar 23;13:4726. doi: 10.1038/s41598-023-31746-x (PMC10036614; doi:10.1038/s41598-023-31746-x)
Supplement: Supplementary file 1 — Supplementary Information. [file 41598_2023_31746_MOESM1_ESM.pdf]

Combining bird tracking data with high-resolution thermal mapping to identify microclimate refugia

Rita F. Ramos, Aldina M.A. Franco, James J. Gilroy, João P. Silva

SI1. Little bustard GPS location information.

In Supplementary Table 1 are presented the details regarding the Little bustards GPS points. The number of individuals per year relates with individuals that were followed for more than one year. The data were filter so GLMM were done considering only the time (hour) period when microclimate refugia was used by the Little bustard (see methods section). The percentage of GPS points above 25ºC is related with the total number of GPS points. The percentage of GPS points in microclimate refugia above 25ºC is related to the total number of GPS points above 25ºC.

Supplementary Table 1 - Information related with little bustard GPS locations

	**Breeding**	**Post-breeding**
**Num. ind. tracked**	77	68
**Num. years**	11 (2009-2019)	
**Num. ind/year**	103	90
**Total num. GPS locations**	43 500	49 185
**Period of use of microclimate refugia (hour)**	6 - 17	7 – 20
GPS points > 25ºC	15 271 (35.1%)	31 198 (63.4%)
**GPS points > 25ºC in microclimate refugia**	296 (1.9%)	422 (1.4%)

SI2. GLMM summary for predictors of microclimate refugia availability

Supplementary Table 2 – GLMM summary for predictors of microclimate refugia availability, for both breeding and post-breeding seasons

	**Breeding**				**Post-breeding**			
	**Estimate**	**Std. Error**	**z value**	**p-value**	**Estimate**	**Std. Error**	**z value**	**p-value**
Intercept	-2.71	0.25	-10.71	< 0.01	-2.73	0.26	-10.74	< 0.01
MCP 50 longitude	**-1.33**	**0.40**	**-3.34**	**< 0.01**	<u>-0.54</u>	<u>0.30</u>	<u>-1.81</u>	<u>0.07</u>
MCP 50 latitude	**1.40**	**0.35**	**4.03**	**< 0.01**	-0.13	0.24	-0.57	0.57
Land use buffer arboreous	**-0.47**	**0.08**	**-5.73**	**< 0.01**	**0.07**	**0.03**	**2.05**	**0.04**
Land use buffer shrubby	**0.40**	**0.07**	**5.85**	**< 0.01**	**0.10**	**0.03**	**3.55**	**< 0.01**
Median Temperature	0.04	0.05	0.86	0.39	**0.10**	**0.03**	**3.61**	**< 0.01**
Std Temp. buffer	**2.10**	**0.08**	**25.85**	**< 0.01**	**2.01**	**0.05**	**40.64**	**< 0.01**
RAC 300m	**3.36**	**0.11**	**31.02**	**< 0.01**	**3.96**	**0.08**	**48.22**	**< 0.01**

SI3. GLMM summary for predictors of microclimate refugia use

Supplementary Table 3 – GLMM summary for the predictors of microclimate refugia use, for both breeding and post-breeding seasons

	**Breeding**				**Post-breeding**			
	**Estimate**	**Std. Error**	**z value**	**p-value**	**Estimate**	**Std. Error**	**z value**	**p-value**
Intercept	-7.91	0.48	-16.56	< 0.01	-9.12	0.50	-18.26	< 0.01
MCP 50 longitude	**-1.17**	**0.54**	**-2.16**	**0.03**	**-1.26**	**0.50**	**-2.54**	**0.01**
MCP 50 latitude	**1.48**	**0.43**	**3.42**	**< 0.01**	0.43	0.36	1.18	0.24
Land use point: arboreous	**5.72**	**0.51**	**11.25**	**< 0.01**	**6.99**	**0.34**	**20.66**	**< 0.01**
Land use point: shrubby	-0.24	0.99	-0.24	0.81	**6.10**	**0.52**	**11.73**	**< 0.01**
Land use buffer arboreous	**-0.82**	**0.23**	**-3.59**	**< 0.01**	**-2.07**	**0.17**	**-12.35**	**< 0.01**
Land use buffer shrubby	0.20	0.20	1.02	0.31	**-0.64**	**0.14**	**-4.41**	**< 0.01**
Active period	-0.52	0.33	-1.57	0.12	**1.09**	**0.21**	**5.13**	**< 0.01**
Julian day	-0.13	0.18	-0.71	0.48	**0.21**	**0.09**	**2.32**	**0.02**
Median temp. buffer	0.20	0.17	1.19	0.24	**0.31**	**0.09**	**3.49**	**< 0.01**
Std Temp. buffer	**0.79**	**0.12**	**6.57**	**< 0.01**	**1.15**	**0.09**	**13.36**	**< 0.01**
RAC 300m	**1.74**	**0.14**	**12.16**	**< 0.01**	**1.05**	**0.07**	**14.24**	**< 0.01**

SI4. Statistics of distance between consecutive points, in an hourly interval, for each season, for each individual per year

Supplementary Table 4 - Basic statistics of distance between consecutive GPS locations (per hour) for each Little bustard per year, in each season

	**Breeding**	**Post-breeding**
**Grouping**	Ind/year	
**Total nº gps locations**	54998	51407
**Mean distance (m)**	129.75	290.52
**Median distance (m)**	36.72	31.10
**Standard deviation of distance (m)**	417.78	2341.01

SI5. CORINE Land Cover to MODIS type of habitat conversion

Supplementary Table 5 - Match between CORINE Land Cover and MODIS habitat categories, used to generate the habitat raster to run the *microclima* package

CORINE Land Cover categories	MODIS categories
Agro-forestry areas	savannas
Complex cultivation patterns	savannas
Transitional woodland-shrub	savannas
Annual crops associated with permanent crops	short grasslands
Land principally occupied by agriculture, with significant areas of natural vegetation	short grasslands
Natural grasslands	short grasslands
Non-irrigated arable land	short grasslands
Pastures	short grasslands
Permanently irrigated land	cropland
Rice fields	cropland
Vineyards	Open shrubland
Sclerophyllous vegetation	Open shrubland
Broad-leaved forest	Evergreen Broadleaf forest
Fruit trees and berry plantations	Evergreen Broadleaf forest
Olive groves	Evergreen Broadleaf forest
Coniferous forest	Evergreen needleleaf forest
Mixed forest	NA
Airports	NA
Construction sites	NA
Discontinuous urban fabric	NA
Industrial or commercial units	NA

SI6. CORINE Land Cover information

Supplementary Table 6 - CORINE Land Cover categories conversion into four classes of vegetation type (arboreous, herbaceous, shrubby and Other type of habitats of land cover), based on their description (EEA 2018)

CORINE Land Cover categories	Vegetation type
Agro-forestry areas	Arboreous
Annual crops associated with permanent crops	Arboreous
Broad-leaved forest	Arboreous
Coniferous forest	Arboreous
Fruit trees and berry plantations	Arboreous
Mixed forest	Arboreous
Olive groves	Arboreous
Beaches, dunes, sands	Herbaceous
Complex cultivation patterns	Herbaceous
Inland marshes	Herbaceous
Land principally occupied by agriculture, with significant areas of natural vegetation	Herbaceous
Natural grasslands	Herbaceous
Non-irrigated arable land	Herbaceous
Pastures	Herbaceous
Permanently irrigated land	Herbaceous
Rice fields	Herbaceous
Salines	Herbaceous
Salt marshes	Herbaceous
Sparsely vegetated areas	Herbaceous
Water bodies	Herbaceous
Water courses	Herbaceous
Construction sites	Other type of habitat
Continuous urban fabric	Other type of habitat
Discontinuous urban fabric	Other type of habitat
Estuaries	Other type of habitat
Industrial or commercial units	Other type of habitat
Intertidal flats	Other type of habitat
Mineral extraction sites	Other type of habitat
Road and rail networks and associated land	Other type of habitat

CORINE Land Cover categories	Vegetation type
Sea and ocean	Other type of habitat
Sport and leisure facilities	Other type of habitat
Airports	Other type of habitat
Sclerophyllous vegetation	Shrubby
Transitional woodland-shrub	Shrubby
Vineyards	Shrubby

After the simplification of CORINE land covers into 4 classes of vegetation type, the degree of change between CORINE 2012 and CORINE 2018, in the 500 meters surrounding of the Little bustard GPS location, was calculated. Between 2012 and 2018, 99.64% and 95.62% of CORINE land cover categories remained the same for breeding and post-breeding seasons, respectively (Supplementary Table 7). In the breeding season, the conversion was done from herbaceous to shrubby vegetation and from shrubby to herbaceous vegetation with 0.36% and 0.01%, respectively. In the post-breeding season, occurred more changes, from arboreous to herbaceous vegetation in 0.86% of the areas; from herbaceous to arboreous (3.29%) or to Other type of habitats of land cover (0.05%); from shrubby to herbaceous vegetation (0.14%) and from Other type of habitats of land cover to herbaceous vegetation (0.04%) (Supplementary Table 7).

Supplementary Table 7 - CORINE Land Cover changes between vegetations classes from 2012 and 2018 maps

CORINE 2018 / CORINE 2012	Breeding season			Post-breeding season			
	**Arboreous (%)**	**Herbaceous (%)**	**Shrubby (%)**	**Arboreous (%)**	**Herbaceous (%)**	**Shrubby (%)**	**Other (%)**
**Arboreous (%)**	4.04	0	0	4.55	0.86	0	0
**Herbaceous (%)**	0	91.78	0.36	3.29	89.08	0	0.05
**Shrubby (%)**	0	0.01	3.82	0	0.14	0.31	0
**Other (%)**	-	-	-	0	0.04	0	1.68

References

EEA (2018) Updated CLC illustrated nomenclature guidelines. European Environment Agency, Copenhagen, 126 pp.
